# Supplementary material for: Multi-objective computational optimization of human 5′ UTR sequences
Source: Brief Bioinform. 2025 May 25;26(3):bbaf225. doi: 10.1093/bib/bbaf225 (PMC12103902; doi:10.1093/bib/bbaf225)
Supplement: Yamada_supplementary_bbaf225 [file yamada_supplementary_bbaf225.pdf]

Supplementary materials for "Multi-objective computational optimization of human 5′ UTR sequences"

Keisuke Yamada, Kanta Suga, Naoko Abe, Koji Hashimoto, Susumu Tsutumi, Masahito Inagaki,

Fumitaka Hashiya, Hiroshi Abe, and Michiaki Hamada

List of Tables

S1 Hyperparamters 3
S2 Selected 5′ sequences and their predicted scores. 3

List of Figures

S1 Baseline designs of 5′ UTR sequences 4
S2 Model perplexity 5
S3 Experimental evaluation of designed 5′ sequences 5
S4 Comparison of decision-making methods 6

Supplementary Methods

1. Baseline optimizations with a single objective

Two sets of 5′ UTR sequences were evaluated as baselines using single-objective optimization targeting MRL improvement. The first set was designed using Optimus 5-Prime and genetic algorithm [1], as originally described in the development of Optimus 5-Prime [2]. Using the same initial pool (pool 3) as in Fig. 4 of the main text, each sequence in the pool was optimized over 100 rounds via the genetic algorithm, with Optimus 5-Prime serving as the single objective function. After optimization, the 20 5′ UTR sequences with the highest predicted MRL scores were selected and subsequently evaluated for additional metrics, including AGC content, G4 score, and *in vitro* stability. The second set of 5′ UTR sequences was collected from the designs of Castillo-Hair *et al.* [3]. This set included four sequences identified as "High MRL" in their large-scale measurements, eight sequences designed using Fast Seqprop [4], and seven sequences designed using the deep exploration network [5].

2. Construction of IVT templates by PCR

Using plasmid DNA containing the eGFP sequence [2], IVT templates were prepared through PCR using the KOD-Plus-Neo kit (Toyobo). The sequence of the templates (952 bp) is shown below. The templates included a T7 promoter (underlined), a specific 50-nt 5′ sequence (N_{50}), the eGFP coding sequence (bold), and a poly-A_{70} tail.

5′ TTATCGAAATTAATACGACTCACTATAGGGACATCGTAGAGAGTCGTACTTA[N_{50}] **ATGGGCGA
ATTAAGTAAGGGCGAGGAGCTGTTCACCGGGGTGGTGCCCATCCTGGTCGAGCTGGA
CGGCGACGTAAACGGCCACAAGTTCAGCGTGTCCGGCGAGGGCGAGGGCGATGCCAC
CTACGGCAAGCTGACCCTGAAGTTCATCTGCACCACCGGCAAGCTGCCCGTGCCCTGG
CCCACCCTCGTGACCACCCTGACCTACGGCGTGCAGTGCTTCAGCCGCTACCCCGACCA
CATGAAGCAGCACGACTTCTTCAAGTCCGCCATGCCCGAAGGCTACGTCCAGGAGCGC
ACCATCTTCTTCAAGGACGACGGCAACTACAAGACCCGCGCCGAGGTGAAGTTCGAGG
GCGACACCCTGGTGAACCGCATCGAGCTGAAGGGCATCGACTTCAAGGAGGACGGCAA
CATCCTGGGGCACAAGCTGGAGTACAACTACAACAGCCACAACGTCTATATCATGGCC
GACAAGCAGAAGAACGGCATCAAGGTGAACTTCAAGATCCGCCACAACATCGAGGACG
GCAGCGTGCAGCTCGCCGACCACTACCAGCAGAACACCCCCATCGGCGACGGCCCCGT
GCTGCTGCCCGACAACCACTACCTGAGCACCCAGTCCAAGCTGAGCAAAGACCCCAAC
GAGAAGCGCGATCACATGGTCCTGCTGGAGTTCGTGACCGCCGCCGGGATCACTCTCG
GCATGGACGAGCTGTACAAGTTCGAATAA** AGCTAGCGCCTCGACTGTGCCTTCTAGTTGCCA
GCCATCTGTTGTTTG[A_{70}] 3′

In the first PCR, an individual forward (Fw) primer (5′ CATCGTAGAGAGTCGTACTTA[N_{50}]ATGGGCGAA TTAAGTAAGGGC 3′) and a universal reverse (Rev) primer (5′ CAAACAACAGATGGCTGGCA 3′) were used to amplify the sequence containing the eGFP coding region. The PCR was performed in accordance with the conditions recommended by the supplier. The thermal cycling conditions were as follows: 98 °C, 2 min → (98 °C, 10 s → 68 °C, 1 min)× 25 cycles → 68 °C, 5 min. After digestion of the template DNA with SpeedCut Dpn I (Rikaken), the PCR product (851 bp) was purified using the Wizard SV Gel and PCR Clean-Up System (Promega). The second PCR was performed on the first PCR product using a universal forward primer containing a T7 promoter (5′ TTATCGAAATTAATACGACTCACTATAGGGACATCGTAGAGAGTCGTACTTA 3′) and a reverse primer with a poly-dT tail (5′ [T_{70}]CAAACAACAGATGGCTGGCA 3′) using following thermal cycling protocol: 98 °C, 2 min → (98 °C, 10 s → 68 °C, 1 min)× 25 cycles → 68 °C, 5 min. All PCR reactions were analyzed by agarose gel electrophoresis, and the 5′ sequences in the second PCR product were confirmed by Sanger sequencing using the ABI PRISM 3500xL Genetic Analyzer (Center for Gene Research, Nagoya Univ.).

3. Preparation of mRNAs

5′ capped mRNAs were prepared by IVT using a hydrophobic-tagged dinucleotide cap analog, DiPure [6]. The capped mRNAs were transcribed in a reaction mixture consisting of 5 ng/µL template DNA (PCR product), 2 mM NTPs, 2 mM DiPure cap analog, 40 mM Tris-HCl (pH 8.0), 8 mM $MgCl_2$, 2 mM spermidine, 5 mM DTT, 0.002 U/µL pyrophosphatase (prepared in-house) and 10 U/µL T7 RNA polymerase (prepared in-house). After incubation at 37 °C for 2 h, DNase (Takara Bio) was added to the reaction at a final concentration of 1 U/µL, followed by an

additional 15 min incubation at 37 °C. The transcript was recovered by LiCl precipitation, and capped RNA was isolated using ion-paired reversed-phase high-pressure liquid chromatography (IP-RP-HPLC) with a YMC-TriartBio C18 column (YMC corp., 250 × 4.6 mm I.D.) on a Shimadzu Prominence HPLC system (pump, LC-20AD; detector, SPD-M40). Solution_A (100 mM TEAA (pH 7.0) containing 5 % acetonitrile) and Solution_B (100 mM TEAA (pH 7.0) containing 50 % acetonitrile) were used at a flow rate of 1 mL/min. The content of Solution_B was increased from 15 to 30 % over 20 min. The column temperature was maintained at 50 °C. Uncapped and capped mRNA were eluted at 11.5 and 15.0 min, respectively, and fully separated. The collected capped mRNA was irradiated with 365-nm light at 4.0 mW/cm^2 for 10 min to remove the hydrophobic tag (MAX-350 compact xenon light source, Asahi Spectra). The capped RNA was further purified by RP-HPLC under the same conditions described above. The purity of the 18 mRNAs was confirmed by denaturing polyacrylamide gel electrophoresis and a dot blot test using anti-dsRNA J2 antibody (Jena Bio).

4. Translation activity measurement of mRNAs in cultured 293T cells

293T cells (Riken Cell Bank, RCB2202) were cultured in DMEM (Wako) supplemented with 10 % fetal bovine serum (Invitrogen) at 37 °C in a 5 % CO_2 atmosphere. mRNA (100 ng per well) was mixed with Lipofectamine MessengerMAX reagent (Thermo, 0.15 µL per well) in Opti-MEM I Reduced Serum Media (Thermo, 10 µL per well) in a 96-well black cell culture plate. After a 10-min incubation, 293T cells suspended in growth media without phenol red (2.0×10^5 cells/mL, 100 µL per well) were added to the RNA/Lipofectamine mixture in the plate. eGFP expression from the mRNAs was monitored using a Spark Microplate Reader (Tecan) with CO_2 humidity control at 37 °C. Fluorescence (excitation at (485 ± 10) nm, emission at (535 ± 10) nm) was measured at 30-min intervals for 24 hours post-transfection. All values were summed to determine the mRNA expression level.

Supplementary Tables and Figures

Supplementary Table S1: Tuned hyperparamaters of the models.

Hyperparameter	LaMBO-CNN	LaMBO-BERT	LaMBO-DNABERT
Learning rate	0.003	0.003	0.3
Entropy penalty	0.003	0.03	0.3
Mask ratio	0.3	0.3	0.2

Supplementary Table S2: Selected 5′ sequences and their predicted scores. Five sequences were selected as described in the method section of the manuscript. Additionally, three sequences were selected from the initial sequence sets using the same selection process.

Index	Sequence	%AGC	MRL	G4	Stability
CNN#1	CACUAGGAAGAGAAGAGAAAAGAAAAUACACAAAUACAAAGGAGCAAACC	94	7.895	-0.1	-0.417
CNN#2	CAGAGAGAAAGCACACAGGAAGACACAAGGACAAGACGCAGAGGAUCCAA	98	7.654	-0.2	-0.422
CNN#3	CAGACAGCAGUAGAGAGACAGAUCCUAACCCAGAAACACAAACAUCUGCU	88	7.804	-0.2	-0.421
CNN#4	CAAAAAAAGAAAAAGAACCAACAGUUGGACCCCAAAUCCUCUGCAAUACA	88	7.715	-0.1	-0.421
CNN#5	CACAAGGAGUAGAACAAAUUCACACAAGCGAAAGAGUACACUUCACUGAU	84	7.946	-0.2	-0.418
BERT#1	CCGAGAACAAAAACGAAAGAGAGAGAGAACACUGAGCUAAGCUGAGCAAC	94	7.763	-0.1	-0.421
BERT#2	ACAAAAGGACAAAACAGAACAGAGAACAGCAGUCGCAAACAGUAGAUACC	94	7.764	-0.2	-0.419
BERT#3	CUUAAAGGAACAAACGAAAAAGGAAAGGAAGCAAAUCCAUGACCAAGGAC	92	7.761	-0.2	-0.421
BERT#4	CUUAAAACAGCAUCAAAGAGAGAAAAAGAAACGUAACAAACCAUAGAGAC	90	7.678	-0.1	-0.418
BERT#5	ACCGGAGCCACAAACGACAGAGGAAAAAGAAGGAGGCACACAUAGACAAC	98	7.605	-0.2	-0.422
DNABERT#1	AAGAGGAAAAAAAAAAAACAACGAAGAAAAAAAAGAGAGAAAGAGAAUAAA	98	7.906	-0.2	-0.413
DNABERT#2	AAGAAAGAAAAAAAAAAACAAAGAAGAAAACAAAGAGACAAAGAAAAAAAA	100	7.819	-0.2	-0.411
DNABERT#3	GAAAAACACAGAGAAACAAUAGUAGAGGAGCCACAUACAGAACACAAGAG	94	7.911	-0.1	-0.420
DNABERT#4	AAGAGAAACACAAAACAGAGAAACACAAGACAAAGACGAACAGAAGUAAA	98	7.887	-0.2	-0.418
DNABERT#5	AAAGAGACAAAGAGAGAAAAGGGACCAAGAAAACUAAGACGAACAAAACC	98	7.766	-0.1	-0.418
Init#1	AGUUCACCAAAGAAGGAAAUUCACAGCUGCAAAUGAGCACGGCAAACAAA	88	7.441	-0.2	-0.426
Init#2	CCCAAAGCCAAUACGAAGCAACAGUUGGAUCCUAAGUCCUCUGAAAAACA	84	7.581	-0.3	-0.423
Init#3	CCCACCAUUUCGACCAAACGAACAUCAACCAAACAAGACACUGUGUAACA	86	7.159	-0.3	-0.425

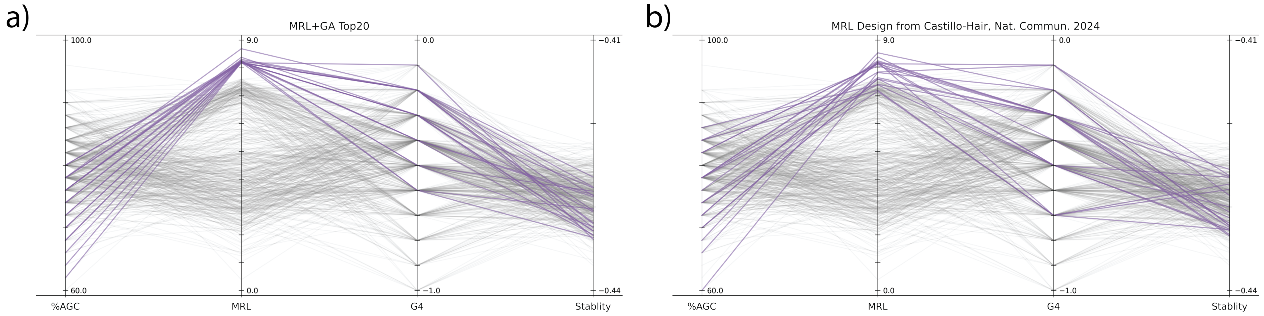

Supplementary Figure S1: In both panels, gray lines represent all 512 sequences in the initial sequence pool (pool 3). a) Colored lines indicate 20 designed 5′ UTR sequences with the highest predicted MRL scores by Optimus 5-Prime, selected from those generated using a genetic algorithm optimized solely for MRL improvement. b) Colored lines indicate 19 designed 5′ UTR sequences (50 nucleotides) reported by Castillo-Hair *et al.* [3].

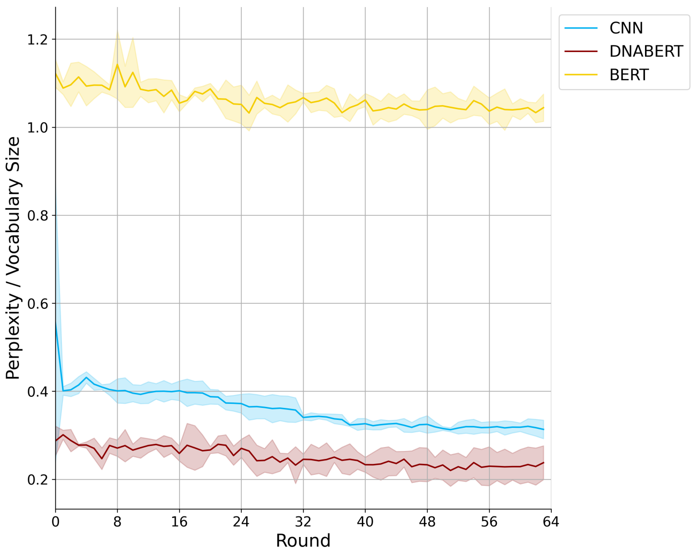

Supplementary Figure S2: Normalized perplexity of each model during training. Perplexity was divided by the vocabulary size of each model, which was 10 for CNN and 69 for BERT and DNABERT.

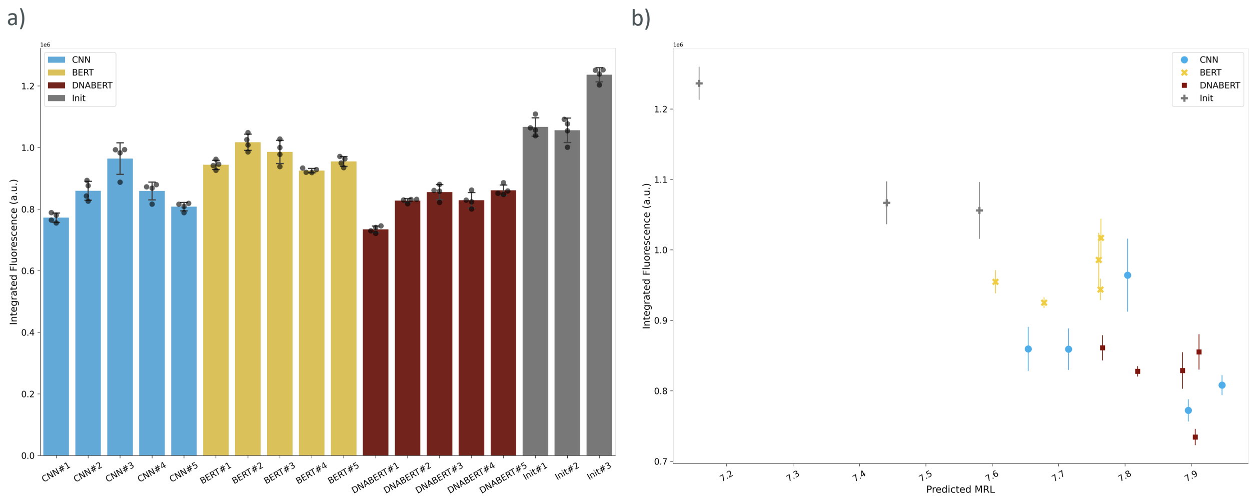

Supplementary Figure S3: The designed 5′ sequences were concatenated with an eGFP coding sequence, transfected into HEK293T cells, and GFP fluorescence was measured to evaluate translation levels. a) Fluorescence was measured over a 24-hour period post-transfection and summed over time. Each dot represents a single data point, and error bars indicate the mean and standard deviation across four replicates. The x-axis labels correspond to those in Supplementary Table S2. b) Relationship between the predicted MRL by Optimus 5-Prime and the measured fluorescence. Each marker represents the mean value, with error bars indicating the mean and standard deviation across four replicates as in a).

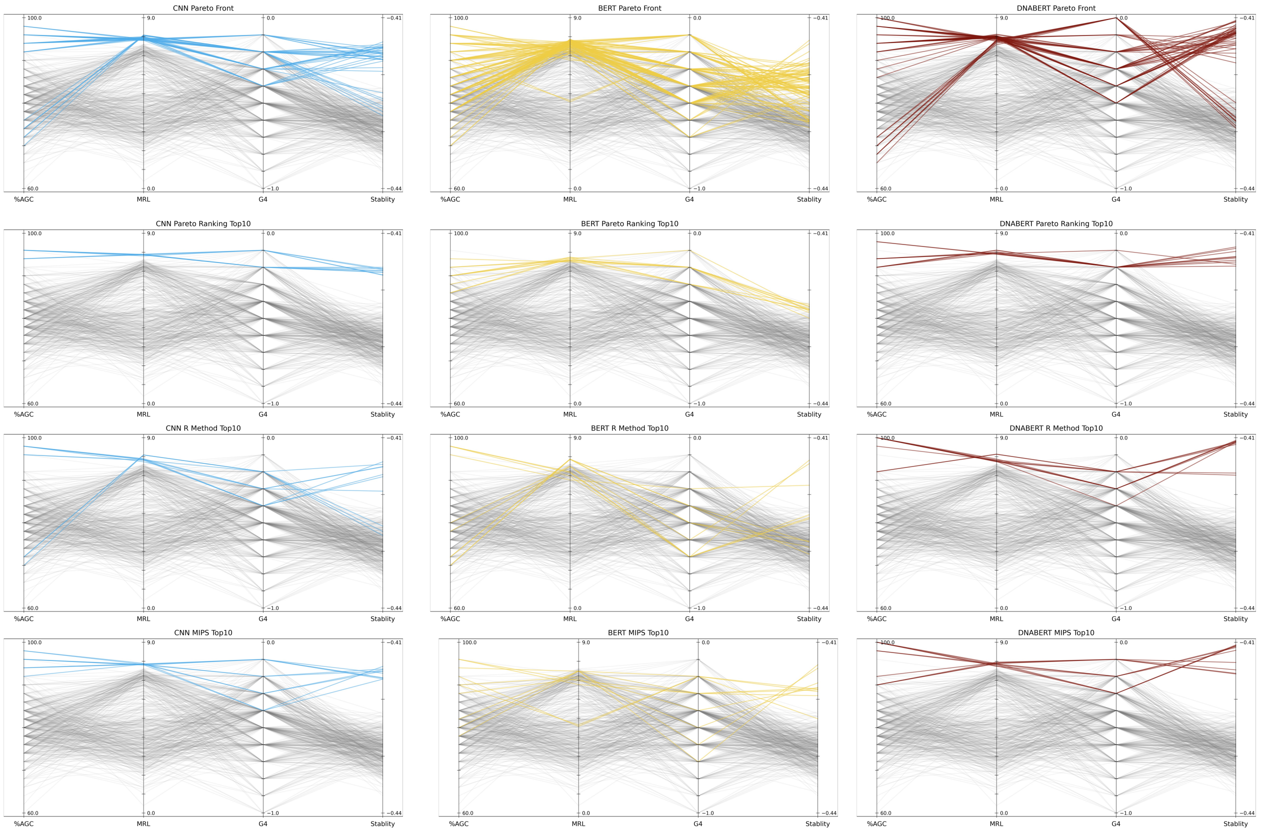

Supplementary Figure S4: Comparison of decision-making methods, including Pareto optimal solutions, Pareto-ranking method, R-method, and MIPS scoring. Gray lines in both panels represent all 512 sequences in the initial sequence pool (pool 3). Colored lines represent the selected candidates based on each decision-making method.

References

[1] Holland, John H. Genetic Algorithms. *Scientific American*. 1992;**267**(1):66–73.

[2] Sample, Paul J *et al.* Human 5′ UTR design and variant effect prediction from a massively parallel translation assay. *Nature biotechnology*. 2019;**37**(7):803–809.

[3] Castillo-Hair, Sebastian *et al.* Optimizing 5′ UTRs for mRNA-delivered gene editing using deep learning. *Nature communications*. 2024;**15**(1):5284.

[4] Linder, Johannes and Seelig, Georg. Fast activation maximization for molecular sequence design. *BMC bioinformatics*. 2021;**22**(1):510.

[5] Linder, Johannes *et al.* A Generative Neural Network for Maximizing Fitness and Diversity of Synthetic DNA and Protein Sequences. *Cell systems*. 2020;**11**(1):49–62.e16.

[6] Inagaki, Masahito *et al.* Cap analogs with a hydrophobic photocleavable tag enable facile purification of fully capped mRNA with various cap structures. *Nature communications*. 2023;**14**(1):2657.
